# Editorial: Gut microbiome and metabolic physiology

**DOI:** 10.3389/fphys.2023.1216411

**Published:** 2023-05-16

**Authors:** Jeanne A. Ishimwe, Annet Kirabo

**Affiliations:** ^1^ Division of Clinical Pharmacology, Department of Medicine, Vanderbilt University Medical, Nashville, TN, United States; ^2^ Center and Department of Molecular Physiology and Biophysics, Vanderbilt University, Nashville, TN, United States

**Keywords:** microbiome, obesity, endocrinology, immune system, inflammation, metabolic disorders, cardiovascular disease, gut-brain axis

**FIGURE 1:**
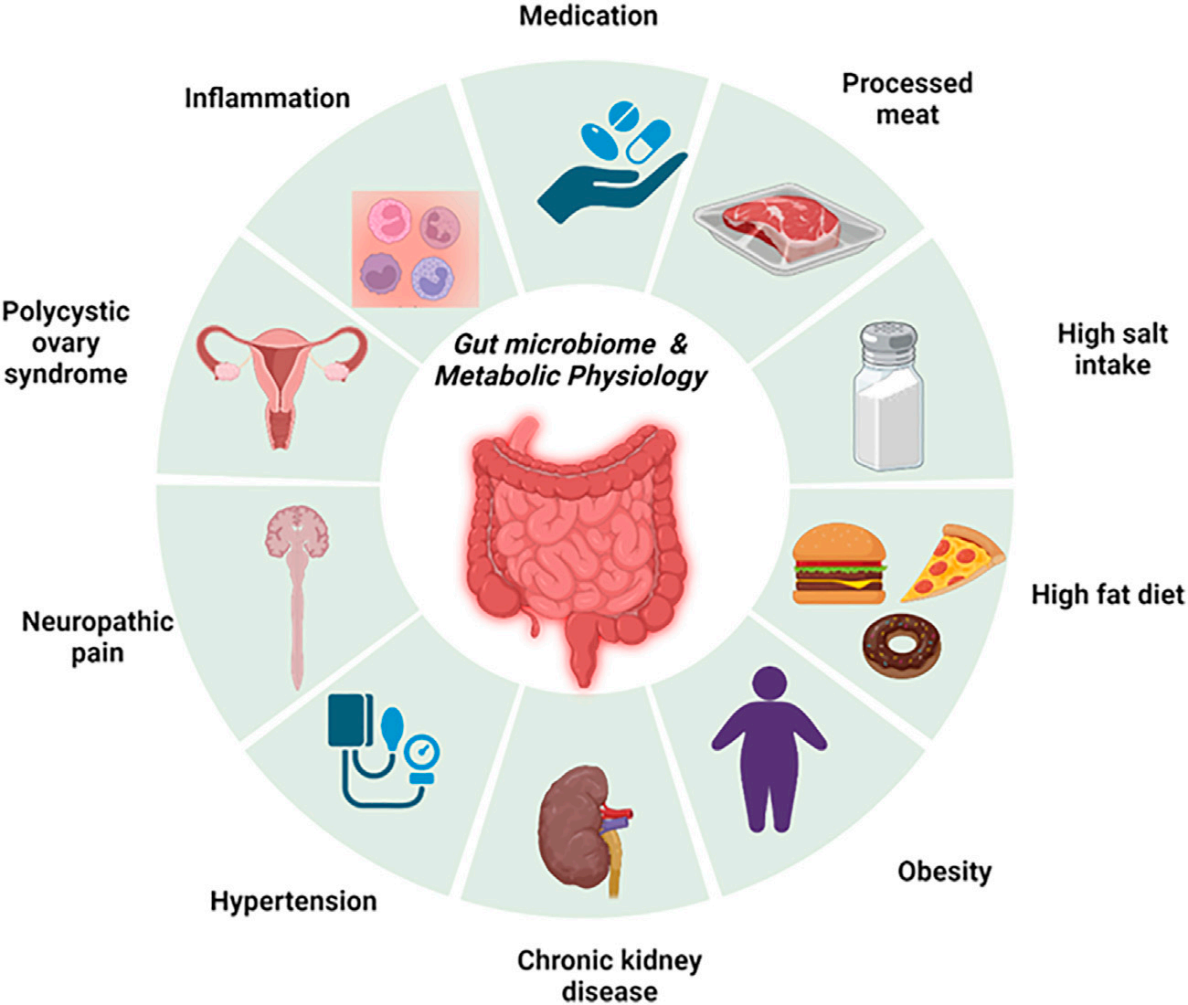
An overview of the gut microbiome and metabolic physiology. This figure summarizes the key chronic cardiometabolic diseases that were investigated in this issue, namely, polycystic ovary syndrome, hypertension, chronic kidney disease, and obesity. In addition, this also highlights key contributors to the development of gut microbiome dysbiosis and impairment of cardiometabolic physiology. These factors include the consumption of high-fat and/or high-salt diets and processed meat.

Gut health is intricately linked with metabolic regulation and physiology. Many factors that drive metabolic changes including dietary patterns like consumption of high salt and/or high-fat diets have a direct effect on the gut (Hasan and Yang, 2019). Despite the widely reported associations between the gut microbiome and cardiometabolic diseases, many gaps remain in the mechanistic links. This Research Topic focuses on the Research Topic “*Gut Microbiome and Metabolic Physiology*.” The goal of the Research Topic was to highlight studies related to microbiome dysbiosis, metabolism, inflammation, the gut-brain axis, and related chronic diseases (Figure 1). It features 4 manuscripts, highlighted below, that review and investigate novel translational concepts in rodents and humans.

Chronic kidney disease affects 37 million American adults and millions more are at risk for developing the disease due to risk factors including diabetes, heart disease, obesity, and hypertension (Kovesdy, 2011). These risk factors share a metabolic dysfunction component in their pathophysiology. Wang et al., explored the link between gut microbiota and chronic kidney disease in association with metabolic dysregulation. The study investigated whether finasteride, a competitive and specific inhibitor of type II 5a-reductase, reduces microbiota-derived trimethylamine N-oxide (TMAO) and alleviates high-fat diet effects in mice with proteinuric nephropathy. TMAO is produced in the liver from trimethylamine which is a gut-derived metabolite of bacterial fermentation of lecithin, choline, L-carnitine, and betaine. The conversion of trimethylamine to TMAO is catalyzed by flavin-containing monooxygenase 3 (Fmo3). Finasteride downregulated Fmo3 and circulating TMAO. Treatment with finasteride maintains tight junction integrity by elevating proteins Claudin-1 and zonulin-1. Finasteride alleviated high fat-associated protein-overload nephropathy and may be a novel therapeutic option to mitigate the pathophysiological relationship between hyperlipidemia and chronic kidney disease progression.

The gut communicates with other parts of the body through the many reported axes including the brain. Diseases that affect the central nervous system are associated with gut microbiota dysbiosis (Ma et al., 2019). Li et al., used osthole, a coumarin compound extracted from the natural product *Angelica biserrata Yuan et*, to target the gut microbiota for the treatment of neuropathic pain. Neuropathic pain was modeled in mice through the fifth and sixth lumbar vertebrae chronic constriction injury (CCI) of the sciatic nerve. They found that CCI was associated with significant changes in the gut microbiota compared to sham mice. Neuropathic pain was associated with higher Bacteriodetes and lower Firmicutes and Verrucomicrobia. Treatment with osthole shifted the gut microbiota compositions to phyla abundance similar to sham mice. Additionally, osthole decreased genera *Bacteroides* and *Akkermansia* while others like *Lactobacillus* were increased. Gut microbiota dysbiosis in neuropathic pain mice was accompanied by elevated glycerolipids, sphingolipids, glycerophospholipids, and fatty acyls which were reversed by osthole treatment. The findings in this study provide preclinical evidence that targeting the gut microbiota may provide an avenue to develop new strategies for the treatment of neuralgia. Specifically, osthole may be a novel therapeutic for neuropathic pain by modulating the gut microbiota and metabolites.

The research studies aforementioned show striking associations between the gut microbiota, metabolome, and disease. It is equally important to note that these mechanisms are not always changed in disease settings. The work by Tayachew et al., examining the effects of combined oral contraceptives on the gut microbiome and metabolome in obese girls with PCOS illustrates this well. The study enrolled 29 participants who were of the female sex, between 12 and 20 years of age, and overweight/obese. Girls were classified as having PCOS if they had oligomenorrhea, clinical/biochemical signs of hyperandrogenism with a minimum of 2 years post menarche based on the National Institute of Health criteria. 8 of the 29 participants were on combined oral contraceptives. The gut microbiome alpha and beta diversity were similar between groups. The study reports differences in the genus *Pseudobutyrivibrio* as the only difference in the gut microbiota between groups. Interestingly, the metabolomic principal component analysis showed distinct clustering by the group despite similar gut microbiota. This difference is said to be largely driven by amino acids, especially tyrosine decreases in the group receiving oral contraceptives. These findings suggest that combined oral contraceptives are not associated with changes in the gut microbiota in obese girls with polycystic ovary syndrome.

As more mechanistic evidence emerges, the current understanding of gut microbiota-dependent pathways such as TMAO in hypertension is becoming clearer (Mutengo et al.). Nearly half of the adults in the United States have hypertension, but it is unfortunately only controlled in a quarter of those affected (Whelton et al., 2022). Elevated TMAO correlates with high blood pressure (Brunt et al., 2021). TMAO promotes hypertension by enhancing angiotensin II-induced vasoconstriction. TMAO also increases oxidized low-density lipoprotein deposition in tissues by disrupting reverse cholesterol transport (Mutengo et al.). Other mechanisms include cardiac mitochondrial and renal dysfunction inflammation. TMAO has been consistently shown to play a pathological role in diseases such as hypertension, obesity, and diabetes. As new mechanisms by which the gut microbiota regulates metabolic physiology, novel therapeutic avenues for related diseases emerge. For example, TMAO production can be blocked by TMA-Lyase inhibitor iodomethylcholine (Gupta et al., 2020). Other pharmacological ways to modulate TMAO levels include phytochemicals and FDA-approved drugs like aspirin and metformin (Iglesias-Carres et al., 2021) making it an attractive target for modulating the gut microbiome dysbiosis in metabolic dysregulation and beyond.

Metabolic syndrome encompasses high blood pressure, atherogenic dyslipidemia, and elevated fasting glucose. This cluster of proatherogenic and proinflammatory states greatly increases the risk for heart disease, stroke, and type 2 diabetes (Lee et al., 2020; Peh et al., 2022). Research presented in this issue shows the role of the gut microbiome in diseases such as hypertension, obesity with polycystic ovary syndrome, kidney disease, and neuropathic pain-associated metabolic disorder. Elucidation of the mechanism of the gut microbiome dysbiosis, its effect on metabolites, and metabolic physiology will lead to novel drugs to treat chronic cardiometabolic diseases.
